# Image‐domain shading correction for cone‐beam CT without prior patient information

**DOI:** 10.1120/jacmp.v16i6.5424

**Published:** 2015-11-08

**Authors:** Qiyong Fan, Bo Lu, Justin C. Park, Tianye Niu, Jonathan G. Li, Chihray Liu, Lei Zhu

**Affiliations:** ^1^ Department of Radiation Oncology University of Nebraska Medical Center Omaha NE USA; ^2^ Department of Radiation Oncology University of Florida Gainesville FL USA; ^3^ Sir Run Run Shaw Hospital Zhejiang University School of Medicine Hangzhou Zhejiang China; ^4^ Institute of Translational Medicine Zhejiang University Hangzhou Zhejiang China; ^5^ Nuclear & Radiological Engineering and Medical Physics Programs, The George W. Woodruff School of Mechanical Engineering Georgia Institute of Technology Atlanta GA USA

**Keywords:** scatter correction, shading correction, CBCT

## Abstract

In the era of high‐precision radiotherapy, cone‐beam CT (CBCT) is frequently utilized for on‐board treatment guidance. However, CBCT images usually contain severe shading artifacts due to strong photon scatter from illumination of a large volume and non‐optimized patient‐specific data measurements, limiting the full clinical applications of CBCT. Many algorithms have been proposed to alleviate this problem by data correction on projections. Sophisticated methods have also been designed when prior patient information is available. Nevertheless, a standard, efficient, and effective approach with large applicability remains elusive for current clinical practice. In this work, we develop a novel algorithm for shading correction directly on CBCT images. Distinct from other image‐domain correction methods, our approach does not rely on prior patient information or prior assumption of patient data. In CBCT, projection errors (mostly from scatter and non‐ideal usage of bowtie filter) result in dominant low‐frequency shading artifacts in image domain. In circular scan geometry, these artifacts often show global or local radial patterns. Hence, the raw CBCT images are first preprocessed into the polar coordinate system. Median filtering and polynomial fitting are applied on the transformed image to estimate the low‐frequency shading artifacts (referred to as the bias field) angle‐by‐angle and slice‐by‐slice. The low‐pass filtering process is done firstly along the angular direction and then the radial direction to preserve image contrast. The estimated bias field is then converted back to the Cartesian coordinate system, followed by 3D low‐pass filtering to eliminate possible high‐frequency components. The shading‐corrected image is finally obtained as the uncorrected volume divided by the bias field. The proposed algorithm was evaluated on CBCT images of a pelvis patient and a head patient. Mean CT number values and spatial non‐uniformity on the reconstructed images were used as image quality metrics. Within selected regions of interest, the average CT number error was reduced from around 300 HU to 42 and 38 HU, and the spatial nonuniformity error was reduced from above 17.5% to 2.1% and 1.7% for the pelvis and the head patients, respectively. As our method suppresses only low‐frequency shading artifacts, patient anatomy and contrast were retained in the corrected images for both cases. Our shading correction algorithm on CBCT images offers several advantages. It has a high efficiency, since it is deterministic and directly operates on the reconstructed images. It requires no prior information or assumptions, which not only achieves the merits of CBCT‐based treatment monitoring by retaining the patient anatomy, but also facilitates its clinical use as an efficient image‐correction solution.

PACS number(s): 87.57.C‐, 87.57.cp, 87.57.Q‐

## I. INTRODUCTION

Modern external beam radiation therapy relies heavily on image‐guided radiation therapy (IGRT) to improve the efficacy and outcome of cancer treatment. Among the many applications of IGRT, gantry‐mounted cone‐beam computed tomography (CBCT) is being increasingly utilized for treatment guidance. It provides three‐dimensional (3D) patient anatomical information at the treatment time as on‐board guidance for processes such as treatment setup and target localization. CBCT is also utilized in more demanding applications such as dose calculation and tumor delineation for adaptive radiation therapy.^1^, ^2^, ^3^ The effectiveness of these CBCT utilizations is closely dependent on the CBCT image quality. Unfortunately, unlike CT with fan‐beam geometry, CBCT images suffer from large shading artifacts which can substantially hinder their applications.

The shading artifacts in CBCT images are caused by several nonidealities. Among them, scatter contamination due to large illumination size (i.e., cone beam geometry) is most severe. For CBCT systems without correction, the scatter‐to‐primary ratio is typically ∼2 on a midsized volume and can be up to 5 on a human torso.^(4)^ Scatter signals are mostly low‐frequency in data acquisition domain (referred to as projection domain),^5^, ^6^ leading to dominant low‐frequency shading artifacts in image domain. The low‐frequency image errors also come from other nonidealities including beam hardening effects, nonoptimized measurement conditions (e.g., limited selection of bowtie filters), and nonideal detector response (e.g., detector lag and nonlinear detector gains). Without effective shading correction, CBCT images suffer from significant CT number errors and severe spatial nonuniformity, limiting the full potential of CBCT imaging in IGRT.^(7)^

There are two major categories of correction methods in the literature to improve the CBCT image quality: preprocessing and postprocessing. Preprocessing methods are mainly hardware‐based and typical examples include the air‐gap,^(8)^ bowtie filter^(9)^ and anti‐scatter grid.^(10)^ While these add‐on devices are able to prevent a certain amount of scattered photons from reaching the detectors, primary photons are attenuated more as well, resulting in patient dose increase if the same signal‐to‐noise ratio is to be maintained. Postprocessing methods correct the scatter artifacts by estimating or measuring scatter in projection domain. Typical approaches include analytical modeling,^11^, ^12^ Monte Carlo (MC) simulations,^13^, ^14^ measurement‐based methods,^7^, ^15^, ^16^, ^17^ and modulation methods.^4^, ^18^, ^19^ Analytical methods usually model the scatter distribution using convolution of the primary fluence with the scatter kernel.^(20)^ The kernel is usually assumed to be shift‐invariant and linear to ensure computational efficiency, which compromises the estimation accuracy, especially for heterogeneous and complex objects. MC methods provide accurate kernel estimation at the price of intensive computation and thus are usually combined with analytical approaches for practical implementation.^(14)^ Measurement‐based methods obtain scatter samples in the scatter‐only regions through the use of radio‐opaque inserts between the X‐ray source and the object and then estimate the full scatter distribution using interpolation/extrapolation. Such approaches often require two scans per projection to compensate for the primary loss and thus can result in almost doubled patient dose. Modulation methods decompose the primary and scatter signal in the Fourier domain (referred to as frequency domain) based on their different responses to a primary modulator inserted into the system, and optimization of this method is still in progress.^(4)^ Readers are referred to the work by Niu and Zhu^(21)^ for a more detailed review of scatter correction methods.

Recently, the use of prior information such as planning computed tomography (pCT) images has been investigated for scatter estimation. Niu et al.^22^, ^23^ proposed to estimate the low‐frequency scatter in the projection domain by forward‐projecting the spatial‐registered pCT images followed by a smoothing process. This method usually generates better image quality but requires intensive computation. Brunner et al.^(24)^ designed a prior image constrained scatter correction algorithm based on the empirical hypothesis that CBCT images can be written as a weighted summation of basis images. Although shading artifacts mitigation has been shown on phantom studies, the underlying hypothesis is still subject to discussion and clinical studies are further needed to demonstrate the effectiveness of the method. Marchant et al.^(25)^ proposed the use of pCT images to estimate the low‐frequency scatter error in the image domain. However, this method introduces geometric mismatch because it forces the estimated shading field to have the same geometric pattern as in pCT images. Li et al.^(26)^ proposed an image‐domain‐based correction algorithm that showed certain effectiveness; however, it requires a prior assumption that CBCT images possess piecewise property, which is often not satisfied for complex clinical patient datasets. The algorithm also relies on optimizations where global minimum is not guaranteed.

The approaches discussed above aim to address the correction using external helpers (i.e., by introducing additional procedures or information such as analytical/Monte Carlo models, measurements using blockers, modulation‐decomposed scatter, and prior patient information or assumption). Although successes have been demonstrated in suppressing shading artifacts using these methods, the associated cost (e.g., increased computational complexity, inconvenient system hardware modifications, or compromised applicability) prevents them from wide clinical use as a standard and efficient correction scheme. In addition, residual shading artifacts may still exist after any or a combination of the above approaches.

In this paper, we propose a shading correction algorithm that is performed in image domain (i.e., operates directly on CBCT images) and requires no prior images or assumptions. Distinct from other existing methods, our approach excludes the use of any external helpers. The algorithm is mainly based on the fact that the uncorrected CBCT volume itself already contains information about both the true patient anatomy and the shading artifacts. The major component of our shading correction method is to decompose or estimate the shading field (referred to as bias field^(26)^ in the following description) from the uncorrected CBCT volume by making use of the intrinsic low‐frequency nature of shading artifacts in image space. With only low‐frequency shading bias field removed from the original CBCT image, the original patient anatomical information and image contrast at the treatment time can be retained, preserving the merits of treatment guidance (e.g., for patient setup) using CBCT images and also allowing more demanding applications (e.g., for dose calculation in adaptive radiation therapy). Since there is no prior information involved and the correction is done in image domain directly, this method does not require intense computation and is deterministic. It fits into current radiation therapy workflow without increase of scan time or radiation dose, without modifications to the conventional imaging hardware or protocols, and without complicated physical modeling. It could be readily incorporated as a "plug‐and‐play" component into the CBCT scanner software and used as a general and standard post‐processing correction solution.

## II. MATERIALS AND METHODS

### A. Algorithms

**Figure 1:**
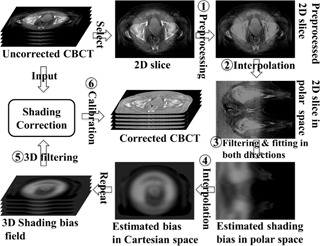
Workflow of the shading correction scheme in image domain without prior information. The shown CBCT images are from the evaluated pelvis patient case. Note that for simplicity, this workflow does not contain the precorrection round that precorrects the global radial pattern.

A workflow that illustrates the major components of our proposed algorithm is shown in Fig. 1.

The algorithm performs correction, slice by slice, directly on the original CBCT volume. For every given 2D transverse slice, the image was firstly centered and then a preprocessing step (Fig. 1, step 1) was performed to prevent the bone, gas regions, and possible fiducial inserts (mostly high‐frequency spatial components) from hampering the estimation of low‐frequency bias field. The pixels of these structures were first identified based on fixed image‐value thresholds and image processing techniques (i.e., image erosion and dilation) and then filled with constant image values of water.

**Figure 2:**
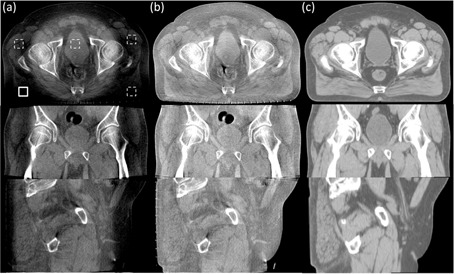
The axial, coronal, and sagittal comparison views of the prostate patient images. Display window: [−250 250] HU: (a) CBCT without correction; (b) CBCT with the proposed shading correction; (c) registered pCT. In the selected uniform soft‐tissue ROI (marked with a solid white square in (a)), the average CT numbers from (a) to (c) are −252 HU, 10 HU, and 52 HU, respectively. The SNUs calculated on the selected five ROIs (marked with solid and dashed white squares in (a)) are 21.2%, 3.2%, and 1.1%, respectively.

**Figure 3:**
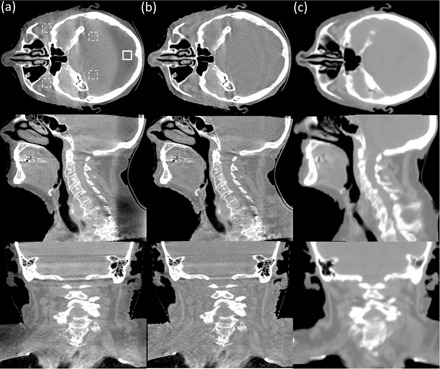
The axial, coronal, and sagittal comparison views of the head‐patient images. Display window: [−500 500] HU: (a) CBCT without correction; (b) CBCT with the proposed shading correction; (c) registered pCT. In the selected uniform soft‐tissue ROI (marked with a solid white square in (a)), the average CT numbers from (a) to (c) are −245 HU, 9 HU, and 47 HU, respectively. The SNUs calculated on the selected five ROIs (marked with solid and dashed white squares in (a)) are 18.8%, 3.0%, and 1.3%, respectively.

In circular scan geometry, shading artifacts often show global or local radial patterns. In fact, the shading can be roughly classified into three types based on their patterns: global radial (largely uniform, as shown in uncorrected pelvis‐case images, see Fig. 2(a)), local radial (as depicted in uncorrected head‐case images, see Fig. 3(a)), and nonradial. Motivated for efficient and convenient estimation of radial‐pattern shading, the preprocessed image was transformed into the polar coordinate system using spline interpolation (Fig. 1, step 2). After conversion, the air pixels outside the patients were first identified, angle by angle, along the radial direction based on the location of maximum image‐value gradient. Those pixels were then flagged so as to be absent from any further processing in order not to compromise the estimation of bias field.

**Figure 4:**
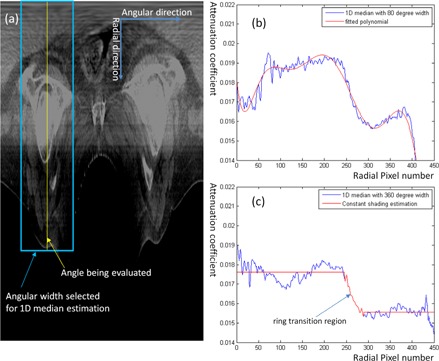
1D median filtering in angular direction: (a) shows that for every angle, the 1D median is calculated as the median of a data array specified by angular width centered at the angle being evaluated; (b) shows the calculated 1D median with 80° width and the estimated shading (i.e., fitted polynomial); and (c) shows the similar 1D shading estimation in the precorrection round designed for shading of global radial pattern.

With the 2D slice of CBCT volume converted into the polar coordinate system, a median filtering step (i.e., the calculation of median, Fig. 1, step 3) was applied along angular direction with a fixed angular width covering the filtering range for every single angle (Figs. 4(a) and 4(b)).

Specifically, the 1D median of the angle being evaluated was firstly calculated from the image range defined by the fixed angular width, as in Fig. 4(a). Then the low‐frequency component of this 1D median was estimated using 1D polynomial fitting. This fitted polynomial forms the bias field of current angle in the polar coordinate system. After operations on all the angles sequentially, the fitted polynomials were concatenated into an initial bias field. The initial bias field then underwent a similar operation for radial direction with a different median filtering width and a different order for polynomial fitting. For the angular‐direction polynomial fitting, the order was set to be 8, while, for radial direction, the order was set to be 3. These numbers were chosen empirically. The resulting bias field, obtained in the polar coordinate system, was then interpolated back into the Cartesian coordinate system (Fig. 1, step 4).

Repeating the above steps, the 3D shading bias field was obtained after such slice‐by‐slice operations followed by a subsequent 3D low‐pass filtering (i.e., 3D median filtering, Fig. 1, step 5) to remove possible high‐frequency components. The corrected CBCT was obtained through dividing the uncorrected CBCT by the shading bias field,

(1) $$I=\frac{I_{0}}{B}$$

where $I_{0}$ represents the original CBCT, *B* represents the estimated bias field, and *I* represents the corrected CBCT. A subsequent calibration, where the corrected CBCT is firstly scaled by the linear attenuation coefficient of water and then converted into CT number, is finally performed (Fig. 1, step 6).

To improve the performance for cases with global radial‐pattern shading (e.g., the pelvis case in Fig. 2(a)), a precorrection round was introduced. This round is typically desired for imaging of large‐size sites (e.g., pelvis, where the half‐fan scan mode is used to increase the field of view) for CBCT systems without an inherent scatter/shading correction algorithm. In this case, with the use of a bowtie filter, there was an obvious ring shading pattern and the shading was largely uniform outside the ring. As indicated by Fig. 4(c), the sharp intensity drop at the ring transition region indicated the largely uniform shading that needs to be compensated for. This precorrection round was formulated to reduce such largely uniform shading to provide both convenience and accuracy for further removal of residual scattered local shading.

The precorrection round resembles the one shown in Fig. 1, except that in step 3 (i.e., the calculation of 1D median) all angles share the same 1D median calculated from a full angular width (i.e., 360° range) and the bias was estimated as described in Fig. 4(c) rather than by polynomial fitting. Specifically, the bias in the inner and outer ring was its mean value of the above calculated 1D median, respectively, while the transition area was kept unchanged. This estimated bias formed the precorrection bias field and it was then converted back into the Cartesian coordinate system based on the same spline interpolation. Finally, the raw CBCT image was divided by this bias field to achieve a precorrection of global radial pattern, as in Eq. (1).

### B. Evaluations

We evaluated our proposed method on a pelvis patient and a head patient. The shading of the pelvis patient presented an obvious global ring pattern and thus the precorrection round was enabled for this patient, though not for the head patient. Two sets of data were available for each case (i.e., the original pCT image and the CBCT projections of one fraction). The pCT scans were taken on a 16‐slice Big Bore CT simulator (Brilliance, Philips Healthcare Systems, Andover, MA) with the helix scan mode, and were used as ground truth for quantitative evaluation. The pCT image had a size of 512×512×102 with a voxel size of 1.17 mm in the axial plane and 3 mm in the longitudinal direction for the pelvis case, and a size of 512×512×84 with a voxel size of 0.683 mm in the axial plane and 3 mm in the longitudinal direction for the head case. The CBCT scans were performed on the patients at the treatment time using a commercial CBCT system (On‐Board Imager, Varian Medical Systems, Palo Alto, CA). The X‐ray voltage and beam quality (in terms of half value layer) is 125 kVp and 6.4 mm Al, 100 kVp and 5.4 mm Al, for pelvis and head cases, respectively. The ring suppression algorithm was not enabled with the CBCT device.

For the pelvis CBCT scan, the system was operated in the half‐fan scan mode with a bowtie filter mounted on the outside of the X‐ray collimator and a data acquisition of 655 projections in a 360° scan. For the head patient, the system was operated in the full‐fan scan mode with a data acquisition of 360 projections in a 200° scan. For both cases, the CBCT images were reconstructed using the standard FDK algorithm with a smoothing filter factor of 0.35. The reconstructed volume had a size of 512×512×190 with a voxel size of 0.977 mm in all directions for the pelvis case and a size of 512×512×240 with a voxel size of 0.683 mm in the axial plane and 0.977 mm in the longitudinal direction for the head case. For both cases, the thresholds for identifying bone and gas/cavity regions, as in Fig. 1, step 1, are >100 HU and [−750 HU, −500 HU], respectively.

To facilitate the quantitative comparison of uncorrected CBCT, corrected CBCT, and pCT images, image registration of pCT image to CBCT image was needed. For the pelvis patient, the rigid registration was performed since it provided sufficiently accurate match for comparison study using selected regions of interest (ROIs). For the head patient, due to relatively large deformation of our dataset, deformable registration was applied to enable accurate comparison. An open‐source software package (3D slicer, www.slicer.org) was used in the registration step for both rigid and deformable registrations. The grid size was set to be 10 and 15 voxels in all directions of the whole image volume for the pelvis and head patients, respectively. The optimization goal was typically reached after approximately 40 iterations.

The 2D image in polar space had a size of 600 pixels (radial direction) by 360 pixels (angular direction). The performance of the algorithm was related to the selection of two relevant parameters: the angular width for 1D median filtering and the order for polynomial fitting. In our current implementation, the radial width was set to be 1 pixel for both cases while the angular width was set to be 80 pixels (i.e., 80°) for the pelvis patient and 40 pixels (i.e., 40°) for the head patient. The polynomial fitting order controls the freedom of low‐frequency shading estimation; lowering the order will suppress the high‐frequency components. In the extreme case of zero order, only constant shading estimation is achieved. As mentioned above, the polynomial fitting order was set to be 8 and 3 for the angular and radial directions, respectively.

In addition to a side‐by‐side image comparison, we used mean CT number errors, with associated standard deviation, and spatial non‐uniformity (SNU) in the selected ROIs, as image quality metrics for quantitative evaluation. The registered pCT image was considered as the ground truth in the comparisons for both metrics. The mean CT number error is defined as the difference between the ground truth and CBCT. The associated standard deviation is defined as the square root of the sum of squares of the standard deviation from both CBCT and pCT.

Low‐frequency errors in the projection data, mostly scatter signals, caused nonuniformity in the reconstructed image. We measured the SNU using a similar definition as in the literature:^(9)^

(2) $$SNU=\frac{{\bar{HU}}_{\text{max}}-{\bar{HU}}_{\text{min}}}{1000}\times 100\%$$

Different ROIs were selected in the CBCT image at both the center and the periphery. ${\bar{HU}}_{max}$ and ${\bar{HU}}_{min}$ are the maximum and the minimum of the mean CT number values of these ROIs, respectively. Note that the true SNU may not be zero. Therefore, the image quality was quantified by the SNU error, which is defined as the difference of SNUs between the CBCT and the ground truth.

## III. RESULTS

### A. Pelvis patient study

Figure 2 shows the axial, coronal, and sagittal comparison views of the CBCT images without correction, with the proposed correction, and the registered pCT images, respectively. Large scatter signals resulted in severe shading artifacts, as shown in Fig. 2(a). Our approach achieved an improved image quality, as shown in Fig. 2(b). The rigid registration yielded a satisfactory geometric match for this patient. The proposed method not only reduced the mean CT number error from 304±32 HU to 42±21 HU in the solid white square as indicated in Fig. 2(a), but also decreased the SNU error from 20.1% to 2.1% within the five selected ROIs. Besides the improvement of image quality, our method was also effective in maintaining image contrast, as can be observed through the comparison of Figs. 2(a), 2(b), and 2(c). It is worth noting that our method faithfully retained the anatomical structures as in the corrected CBCT image, which is one of the advantages of shading correction without prior information.

### B. Head patient study

Figure 3 shows the axial, coronal, and sagittal comparison‐views of the CBCT images without correction, with the proposed correction, and the registered pCT images. Heavy scatter signals resulted in large shading artifacts, as shown in Fig. 3(a). The proposed method achieved a better image quality on this more challenging head case, as shown in Fig. 3(b). The deformable registration ensured acceptable geometric match on this patient. The proposed method not only reduced the mean CT number error from 292±28 to 38±17 HU in the solid white square as indicated in Fig. 3(a), but also decreased the SNU error from 17.5% to 1.7% within the five selected ROIs. Similarly as in the pelvis patient, the image contrast was mostly retained, as can be observed via comparison of Figs. 3(a), 3(b) and 3(c). Besides, the anatomical structures in the head image were also fully preserved without any geometric distortion.

## IV. DISCUSSION

In this work, we proposed to correct for CBCT shading artifacts using an image‐domain method without prior patient information. While excluding the use of prior information can avoid either forced geometric mismatch or increased computation, the absence of such information presents daunting challenges in correctly estimating the bias field from uncorrected CBCT images. Nevertheless, by taking full advantage of the fact that the shading artifacts in the image domain have dominant low‐frequency components originating from low‐frequency scatter in the projection domain, our method equipped with low‐pass filtering components was capable of effectively removing the low‐frequency image errors. The advantages of our method mainly lie in two aspects. First, our algorithm removes artifacts in the image domain directly without any data manipulations in the projection domain so as to avoid intensive computation (e.g., forward projection operations and subsequent image reconstruction as in some approaches^(21)^). Second, the proposed algorithm does not utilize any patient prior information that is often not readily available in current clinical practice. Even when patient prior information including the planning CT images is available, the use of such information complicates the shading correction in various ways, including the increase of computation burden or the introduction of geometrical distortion for on‐board CBCT. Therefore, the shading artifacts can be removed efficiently without any additional cost via the proposed method. This method fits the clinical needs and can be used as a standard and general image‐correction solution. In addition, it is worth emphasizing that our method can not only be used alone, but can also be applied following other correction methods, to further improve image quality.

The algorithm was inspired by the motivation to eliminate global radial‐pattern shading artifacts as in the pelvis patient. However, application of the proposed method is not limited to such situations. In fact, the scatter pattern of CBCT images can be case‐dependent, as demonstrated by the images of the above two patients. This is because scatter effects depend on many factors, including patient size and geometry, treatment site, radiation beam spectrum, detector configurations, scanning technique, and the use of bowtie filters. Our method is designed to remove the low‐frequency shading artifacts regardless of the case‐dependent scatter patterns of the shading. Therefore, the algorithm also works for non‐radial‐pattern cases, as long as the shading is dominantly low‐frequency.

In the current study, the algorithm was implemented in MATLAB (MathWorks, Natick, MA) and the calculation speed was not optimized. Computational efficiency could be improved by converting MATLAB code into more efficient languages (e.g., C/C++). In addition, the algorithm performs correction on the original CBCT volume angle‐by‐angle and slice‐by‐slice, a natural parallel computing structure that suggests further computation acceleration using hardware‐based techniques such as GPU.^(27)^ Furthermore, since the bias field contains only low‐frequency components, the uncorrected image from which the bias field is estimated does not need to be in high resolution. Therefore, it can be downsampled first for processing. The resulted low‐resolution bias field can then be upsampled to calculate the final corrected image.

The choices of the low‐pass filtering parameters, such as median filtering width and polynomial fitting order, may affect the performance of the algorithm. The image contrast could be compromised if parameters are not properly chosen. Currently, these parameters are empirically chosen using a brute‐force search scheme, and therefore can be further optimized. It is also worth noting that although we claim that the shading artifacts are mainly from scatter contamination, the algorithm also removes the low‐frequency shading artifacts resulting from other nonidealities such as beam hardening effects, detector lag, nonlinear detector gains, and the use of bowtie filter. However, infrequent high‐frequency shading artifacts can also exist in the image domain. With only low‐pass filtering designed into this method, the current algorithm has difficulties dealing with scatter‐induced high‐frequency artifacts. Also, it should be noted that our algorithm will generate artifacts when there are ring structures that happen to be centered around the origin. This could potentially be solved by shifting the image slightly away from the origin. Another limitation of our method is that it cannot improve the image contrast although it almost retains the contrast of the original image, simply because the correction is done in the image domain and no prior information is used. To address these two limitations, a further step is needed, which could be accomplished by forward‐projecting the corrected volume into projection space and then removing the errors in projection domain using a low‐pass filtering technique. It should be also pointed out that our proposed method is designed to mainly eliminate the shading artifacts. Other artifacts, such as those caused by metals, are beyond the scope of this work, although the method itself may not be compromised by, for example, metal‐induced artifacts, because such high‐frequency artifacts are almost ignored by the low‐pass filters.

## V. CONCLUSION

In summary, we developed a novel shading correction algorithm in image domain without using any prior patient information but only the properties of shading artifacts. The algorithm is stable and effectively removes the low‐frequency errors in CBCT images. The CT number errors are significantly reduced and the spatial uniformity is substantially improved for both the pelvis and the head patients. The anatomical structures of the patients (mostly high‐frequency) are fully retained in the corrected images. The method could be readily implemented as a general and standard image correction solution for clinical CBCT applications, achieving, for example, more accurate patient setup and dose calculation in adaptive radiation therapy.

## ACKNOWLEDGMENTS

We would like to acknowledge support from the Georgia Institute of Technology new faculty startup fund, NIH Grant R21EB012700, the Natural Science Foundation of China Grant 81201091, and the National High‐tech R&D Program for Young Scientists by the Ministry of Science and Technology of China (Grant 2015AA020917).
